# Extensively drug resistant tuberculosis in Mali: a case report

**DOI:** 10.1186/s13104-017-2890-4

**Published:** 2017-11-06

**Authors:** Bassirou Diarra, Yacouba Toloba, Bakary Konate, Moumine Sanogo, Antieme Combo Georges Togo, Fatimata Camara, Gaoussou Berthe, Dianguina Soumaré, Bocar Baya, Drissa Goita, Yeya dit Sadio Sarro, Mamoudou Maiga, Michael Belson, Susan Orsega, Sounkalo Dao, Robert L. Murphy, Sophia Siddiqui, Bouke C. de Jong, Seydou Doumbia, Souleymane Diallo

**Affiliations:** 1 University Clinical Research Center (UCRC)-SEREFO-Laboratory, University of Sciences, Techniques and Technologies of Bamako (USTTB), Bamako, Mali; 2 Service de Pneumo-phtisiologie du Centre Hospitalier Universitaire du Point-G, Bamako, Mali; 3 Programme National de Lutte Contre la Tuberculose (PNLT), Ministère de la Santé et de l’hygiène Publique, Bamako, Mali; 4 Laboratoire National de Référence des Mycobactéries (LNR), Institut National de Recherche en Santé Publique (INRSP), Bamako, Mali; 5 Global Health, Northwestern University, Chicago, IL USA; 6 Collaborative Clinical Research Branch, Division of Clinical Research, National Institute of Allergy and Infectious Diseases, Bethesda, MD USA; 7 Department of Biomedical Sciences, Institute of Tropical Medicine, Antwerp, Belgium

**Keywords:** XDR-TB, Treatment, Bamako, Mali

## Abstract

**Background:**

Drug resistant tuberculosis presents a major public health challenge.

**Case presentation:**

We present here the first two patients diagnosed with extensively drug resistant tuberculosis in Bamako, Mali. Genotypic findings suggest possible nosocomial transmission from the first patient to the second one, resulting in superinfection of the second patient. After being diagnosed with extensively drug resistant tuberculosis in August 2016, the patients only started receiving appropriate treatment 10 months later.

**Conclusion:**

The identification of these patients highlights the need for improved diagnostic and treatment algorithms for better surveillance and management of drug resistance in Mali. In the interest of these as well as future patients suffering from resistant tuberculosis, all steps recommended for programmatic management of drug resistant tuberculosis must be urgently prioritized in order to strengthen the multidrug resistant tuberculosis program.

## Background

Multidrug-resistant (MDR) tuberculosis is defined as disease caused by *Mycobacterium tuberculosis* complex strains with resistance to, at least, isoniazid and rifampicin, while extensively drug resistant tuberculosis (XDR-TB) is defined as MDR-TB plus resistance to a fluoroquinolone and to a second-line injectable agent [1]. By 2015, 117 World Health Organization (WHO) member states had reported at least one patient with XDR-TB [1]. Out of the 7579 XDR patients worldwide in 2015, 1100 (14.5%) were notified in the Africa region. Underreporting is higher in this part of the world, likely due to limited availability of drug susceptibility testing (DST), especially to second line drugs [1, 2].

Mali reported in 2015 a TB incidence of 57 per 100,000 population and was estimated to have 3.5% of primary rifampicin resistance (RR) among smear positive new patients [1]. In Mali, after the first GeneXpert machine was installed in March 2014, another eight have since been installed throughout the country. GeneXpert is used for the detection of RR in high risk patients, such as those who failed, are lost to follow up, or relapse after completion of the standardized 6 month first line therapy. At the University Clinical Research Center (UCRC) SEREFO Laboratory, DST is offered to selected patients enrolled in research protocols, since the biosafety level 3 (BSL-3) laboratory was opened in 2006 [3]. In addition, per national guidelines, all samples from patients at risk of MDR-TB are sent to UCRC for culture and 1st line DST, while confirmed MDR patients are referred to the TB unit of the University Teaching Hospital of Point-G (UTH), Bamako for further assessment, treatment and hospitalization until smear conversion [3]. Second line DST is conducted at the WHO/supra national laboratory at the Institute of Tropical Medicine (ITM), Antwerp, Belgium. We present here the first two documented patients with XDR-TB identified among the MDR-TB patients in Mali.

## Case presentation

### Description of patients

From 20 MDR-TB or RR patient’s samples shipped to ITM in August 2016, two were confirmed as XDR. Both showed high-level resistance for different anti-tuberculosis drugs (Table 1). Although this was for routine surveillance per national guidelines, patients provided their informed consent.

**Table 1:** Second-line drug susceptibility results for patient 1 and 2

	Sample collection date	MTBDRplus rpoB	MTBDRplus katG	MTBDRplus inhA	MTBDRsld gyrA	MTBDRsld rrs	MTBDRsld eis
Patient 1	May 2016	MUT1 (D516V)	MUT1 (S315T)	MUT3B (T8A)	MUT3C (D94G)	MUT1 (A1401G)	WT
Patient 2	May 2016	MUT1 (D516V)	WT	MUT3B (T8A)	MUT3C (D94G)	MUT1 (A1401G)	WT
Patient 3	Feb 2017	MUT1 (D516V)	MUT1 (S315T)	MUT3B (T8A)	MUT3C MUT3B (D94G)	MUT1 (A1401G)	WT

“Point mutations in: the rpoB gene confer resistance to rifampicin; katG gene and in the inhA gene promoter confer resistance to isoniazid; gyrA and gyrB confer resistance to fluoroquinolones; and rrs confers resistance to kanamycin” WT = wild type (no resistance). MUT = mutation in position 1, position 3B, or position 3C of the gene, with specific mutation indicated

#### Patient 1

Is a 27 year old woman living in Bamako. She was first treated for TB in 2009 [category 1: rifampicin (R), isoniazid (H), pyrazinamide (Z), ethambutol (E): 2RHZE/4RH], achieving cure. In 2013 she relapsed and failed category 2 [rifampicin (R), isoniazid (H), pyrazinamide (Z), ethambutol (E), streptomycin (S): 2RHZES/1RHZE/5RH] treatment. She was diagnosed as human immunodeficiency virus (HIV) infected in 2013, when she started taking Atripla^®^ (efavirenz/emtricitabine/tenofovir). In 2014 she was admitted in the specialized tuberculosis unit of the UTH for chronic tuberculosis and suspicion of drug resistance. At first she was started on second-line treatment empirically in February 2014 [kanamycin (K), ofloxacin (O), ethionamide (Et): 6KOEtZ/15OEtZ], with culture revealing a non-tuberculous mycobacterium (NTM) only. She was recognized as suffering from RR-TB through GeneXpert testing in May 2014, and continued to receive second-line treatment but without any clinical improvement. A sputum culture from June 2015 grew *M. tuberculosis* complex (MTBc), and the sample was sent for second-line testing at ITM in December 2015, where fluoroquinolone resistance was diagnosed. As appropriate and effective drugs for pre-XDR were not available in-country, she was re-admitted in the hospital in December 2015, and treated with a weak treatment regimen consisting of kanamycin, cycloserine, amoxicillin/clavulanic acid, and erythromycin. During the hospitalization, observation of therapeutic compliance was irregular. In August 2016, samples were collected and tested again at ITM, and revealed additional resistance to injectable agents. The patient had a close contact with MDR who had been living with her between 2012 and 2013. He died in June 2014, 18 months after starting a second-line drug regimen (6KOEtZ/15OEtZ). He had returned from Cote d’Ivoire in 2010 with a history of interrupted TB treatment.

#### Patient 2

Is an HIV negative 50 year old woman who was referred from Southern Mali in January 2016 for chronic tuberculosis, with GeneXpert identifying RR. She had first been diagnosed with TB in 2014 and started category 1 treatment (2RHZE/4RH) but did not complete due to stock interruptions at her health center. Her health further deteriorated in 2015 with persistent cough and weight loss, and sputum tested positive for acid fast bacilli (AFB) in July 2015, with chest X-ray showing bilateral disseminated micronodular infiltrates associated with a cavity in the right upper lung. She started MDR treatment in January 2016 (6KOEtZ/15OEtZ) after GeneXpert showed RR. Although the treatment was not directly observed during the first 6 month, there was no treatment interruption, and the patient was discharged in June 2016 after her sputum smear had converted to negative.

**Fig. 1:**
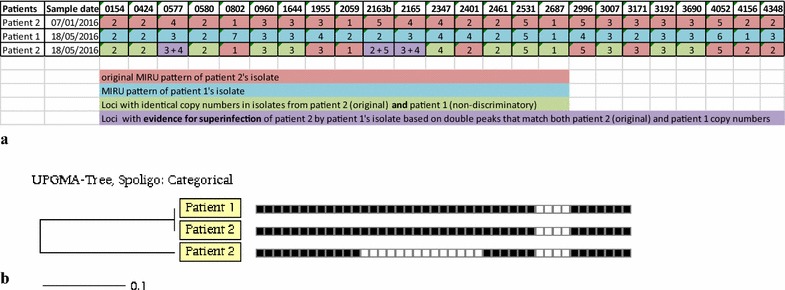
**a** 24-locus mycobacterial interspersed repetitive units, variable number of tandem repeats patterns of both extensively drug resistant tuberculosis patients. Three loci show double peaks for the second sample of patient 2, with the same alleles as patient 1’s MIRU pattern, suggestive of *possible* nosocomial superinfection of patient 2 from patient 1. In addition each copy number in patient 2’s second isolate matches either the original isolate (red = original patient 2, and green = both original patient 2 and patient 1), or is a double pattern with copy numbers matching both original patient 2 AND patient 1 copy numbers. **b** Spoligotyping results of the extensively drug resistant tuberculosis patients: [patient 2, sample 1 (Jan 16) is at the bottom and sample 2 (May 16) is in the middle] showing the superimposed pattern of patient 1 obscuring the missing spacers 13–26 in the first isolate. All isolates belong to the EuroAmerican lineage 4

She had no known close contact with a MDR patient, except for patient 1 described above, with whom she shared the same hospital ward between January and June 2016. Her spoligotype pattern changed from *M. tuberculosis* (MTB) T4 family in January 2016, to MTB T1 family (same as patient 1) in May 2016 (Fig. 1b). Despite both patients having the same MTB T1 spoligotype pattern on cultures isolated in May 2016, by 24 locus mycobacterial interspersed repetitive units, variable number of tandem repeats (MIRU-VNTR) the patterns differed in six loci, while patient 2 had proof of mixed infection defined as double peaks in three of 24 loci (Fig. 1a), with the additional peaks matching the MIRU pattern of patient 1. This suggests that patient 2 had chronic TB with a different strain, but was superinfected by patient 1 through nosocomial transmission. The different resistance profiles [*katG* mutation for patient 1, and wild type (WT) for patient 2] suggest that the Line Probe Assay missed the superinfecting strain in patient 2 (Fig. 1, Table 1). In addition each copy number in patient 2’s second isolate matches either the original isolate (red = original patient 2, and green = both original patient 2 and patient 1), or is a double pattern with copy numbers matching both original patient 2 AND patient 1 copy numbers.

### From diagnosis to treatment initiation

After diagnosis of these patients as XDR in August 2016, in November 2016 the Mali national tuberculosis program (NTP) placed an order for assistance with the Global Drug Facility (GDF) Stop TB Partnership to initiate a treatment regimen with new drugs. Despite two additional requests through The TB Union and also WHO, appropriate treatment was not made available until finally the Global Fund through its representative program in Mali bought the drugs, bedaquiline (Bdq), clofazimine (Cfz), delamanid (Dlm), linezolid (Lzd), para acid salicylic (PAS): 6 (Bdq-Cfz-Dlm-Lzd-Z-H-PAS)/14 (Bdq-Cfz-Lzd-Z) and patients started treatment on May 9th, 2017. Sadly, during this 10 month delay, patients were hospitalized in isolation rooms without receiving TB specific therapy, only nutritional support.

### Actions taken to limit transmission

After diagnosis, in addition to the isolation of the two patients from the other MDR patients, all the household contacts for both patients were screened for TB, including drug resistance, by Xpert MTB/Rif. This screening allowed identifying another MDR-TB patient from patient 2’s family. Unfortunately, at the end of March 2017, we identified a third XDR-TB patient also from the same hospital ward, and molecular Hain test suggest nosocomial transmission from the first XDR patient (Table 1). We think that the most logical explanation is that patient 3 has pre-XDR with a *gyrA* MUT3B, and was superinfected by patient 1, who had XDR with *gyrA* MUT3C. In total three XDR and one MDR patient were identified from this outbreak.

### Culture, identification, first line drug susceptibility testing at SEREFO/UCRC and shipping of isolates to ITM and molecular second-line DST at ITM, Antwerp Belgium

In the UCRC laboratory, primary isolation is done in both liquid [manual reading of *Mycobacterium* growth incubator tubes (BBL™ MGIT™ Becton–Dickinson, Sparks MD, USA)], and solid (Middlebrook 7H11 agar and selective 7H11 agar) media, following standard protocols. Indirect first line DST is performed on confirmed *M. tuberculosis* complex (MTBc) isolates using MGIT AST/SIRE System (Becton Dickinson, Sparks, MD, USA). Samples and cultured isolates were first heat inactivated before shipping to ITM, Antwerp, where the Hain Second Line Probe Assay, (GenoType MTBDRsl) was performed on each sample as per manufacturer’s instructions [4].

## Discussion and conclusions

Data on XDR-TB are scarce in Africa, especially in West African countries [2, 3]. We describe here the first documented XDR patients in Mali. Extensively drug resistant TB patients were identified in neighbouring countries Burkina Faso in 2010 and Cote d’Ivoire in 2015 [5, 6]. As culture and DST were not performed during the first episodes of TB infection in the patients presented here, we cannot exclude primary pre-XDR resistance. Also in the XDR patients in Burkina Faso and Cote d’Ivoire [5, 6] baseline resistance tests were missing, whereas in South Africa primary resistance was well documented in XDR patients [7]. The NTM isolated from the first culture of patient 1 may have been a ‘colonizer’ that obscured ongoing TB disease, or may have contributed to chronic pulmonary infection in this HIV co-infected patient [8]. These patients also highlight the need for new diagnostic tools that could simultaneously detect the MTBc and NTM. In addition, both patients experienced treatment interruptions, and patient 1 was inappropriately treated with a weak regimen based on kanamycin for more than 6 months after diagnosis of high level fluoroquinolone resistance [9], which likely caused the additional resistance to injectables, resulting in XDR-TB. Lastly, the ineffective treatment and poor hospital infection control likely permitted the possible nosocomial superinfection from patient 1 to patient 2 and 3. Urgent treatment initiation limits morbidity, mortality, and ongoing transmission [10, 11]. Here, early initiation of appropriate treatment could have stopped the possible nosocomial transmission and may have prevented the third XDR-TB patient. Despite the resource limited condition with the Mali NTP, it is high time that all recommended steps for programmatic management of drug-resistant tuberculosis (PMDT) implementation for strengthening the MDR TB program are taken to serve patients with rifampicin resistance in Mali.

In order to address the deficiencies presented, firstly the laboratory network in Mali should be upgraded to allow local access to second-line DST, which will considerably reduce the time to diagnosis, especially given the complexities of transportation of samples from Bamako to Antwerp. Both the Hain SL LPA and phenotypic second line DST are currently being implemented not only at the NRL, but also at UCRC. Secondly, patient 2 probably was superinfected by patient 1, and clinicians should be trained for proper management of MDR patients, including appropriate infection control measures, physiotherapy and psychosocial support for patients for retention into care. Most importantly, rapid diagnosis is futile without appropriate treatment regimens being available. As for ‘simple’ MDR patients, the 20 + month MDR regimen has unacceptably high drop-out and failure rates, and the 9M MDR needs to urgently be adopted in Mali. For these and additional XDR-TB patients, appropriate drugs need to be stocked.

In summary, the identification of XDR in Mali is not unexpected, as resistance is a man-made problem and management of patients with MDR-TB is at present not following recommended PMDT guidelines in Mali. We, as the whole management team of MDR-TB patients, failed in multiple steps after the first identification of their extensive resistance, and thus we hope that the measures to be taken will be sufficient to contain resistance TB and prevent its further spread. The lessons learned in Mali may serve as an example to other countries that have not yet identified XDR-TB patients.

## Abbreviations

MDR: multidrug resistant
XDR: extensively drug resistance
WHO: World Health Organization
DST: drug susceptibility testing
RR: resistance to rifampicin
UCRC: University Clinical Research Center
BSL-3: biosafety level 3
UTH: University Teaching Hospital of Point-G
ITM: Institute of Tropical Medicine
DR-TB: drug resistant-tuberculosis
USTTB: University of Sciences, Techniques and Technologies of Bamako
TB: tuberculosis
R: rifampicin
H: isoniazid
Z: pyrazinamide
E: ethambutol
S: streptomycin
K: kanamycin
O: ofloxacin
Et: ethionamide
HIV: human immunodeficiency virus
NTM: non-tuberculous mycobacteria
Mtbc: *Mycobacterium tuberculosis* complex
AFB: acid fast bacilli
MTB T1/T4: *Mycobacterium tuberculosis* T family 1 and 4
MIRU-VNTR: mycobacterial interspersed repetitive units, variable number of tandem repeats
WT: wild type
NTP: National TB Program
DGF: Global Drug Facility
Bdq: bedaquiline
Cfz: clofazimine
Dlm: delamanid
Lzd: linezolid
PAS: para acid salicylic
MUT: mutation
MGIT: mycobacterium growth incubator tube
BD: Becton–Dickinson
MD: Maryland
NIAID/NIH: National Institutes of Allergy and Infectious Diseases, National Institutes of Health
USA: United States of America
AST: antimycobacterial susceptibility testing
SIRE: streptomycin, isoniazid, rifampicin, ethambutol
PMDT: programmatic management of drug-resistant tuberculosis
LPA: Line Probe Assay
NRL: national reference laboratory
IRB: institutional review board
